# *Aspergillus oryzae*–*Saccharomyces cerevisiae* Consortium Allows Bio-Hybrid Fuel Cell to Run on Complex Carbohydrates

**DOI:** 10.3390/microorganisms4010010

**Published:** 2016-02-04

**Authors:** Justin P. Jahnke, Thomas Hoyt, Hannah M. LeFors, James J. Sumner, David M. Mackie

**Affiliations:** Army Research Laboratory, 2800 Powder Mill Road, Adelphi, MD 20740, USA; justin.jahnke2.ctr@mail.mil (J.P.J.); thomas.hoyt@usma.edu (T.H.); hannah.lefors@terpmail.umd.edu (H.M.L.); james.j.sumner4.civ@mail.mil (J.J.S.)

**Keywords:** *Aspergillus oryzae*, mold, *Saccharomyces cerevisiae*, yeast, consortium, bio-hybrid, fuel cell, carbohydrate, FTIR

## Abstract

Consortia of *Aspergillus oryzae* and *Saccharomyces cerevisiae* are examined for their abilities to turn complex carbohydrates into ethanol. To understand the interactions between microorganisms in consortia, Fourier-transform infrared spectroscopy is used to follow the concentrations of various metabolites such as sugars (e.g., glucose, maltose), longer chain carbohydrates, and ethanol to optimize consortia conditions for the production of ethanol. It is shown that with proper design *A. oryzae* can digest food waste simulants into soluble sugars that *S. cerevisiae* can ferment into ethanol. Depending on the substrate and conditions used, concentrations of 13% ethanol were achieved in 10 days. It is further shown that a direct alcohol fuel cell (FC) can be coupled with these *A. oryzae*-enabled *S. cerevisiae* fermentations using a reverse osmosis membrane. This “bio-hybrid FC” continually extracted ethanol from an ongoing consortium, enhancing ethanol production and allowing the bio-hybrid FC to run for at least one week. Obtained bio-hybrid FC currents were comparable to those from pure ethanol—water mixtures, using the same FC. The *A. oryzae–S. cerevisiae* consortium, coupled to a bio-hybrid FC, converted food waste simulants into electricity without any pre- or post-processing.

## 1. Introduction

There has been an increasing interest in developing new methods of extracting energy from biomass to both provide new sources of energy and to remediate waste [1,2]. As a part of their metabolisms, many microorganisms, by themselves or as a part of microbial consortia, can convert biomass in waste streams into chemicals such as methane, hydrogen, and ethanol that are more easily used as fuels [3,4,5]. Both converting the biomass into fuels and extracting the fuels from the waste stream can be challenging. In some cases, this intermediate state of converting biomass into fuel can be skipped by using microbes to directly produce electricity, as in microbial fuel cells (MFCs), where microbes use an electrode as an electron source to oxidize organic matter. MFCs are capable of digesting many substrates and directly producing electrical energy with high coulombic efficiencies [6], but have been hampered by their low power densities, despite continued progress at improving electron transfer between electrodes and microorganisms [7,8]. Another promising technology is enzymatic FCs, which typically utilize immobilized enzymes as oxidation catalysts. However, as pointed out in [7], FCs utilizing whole organism catalysts are more robust than enzymatic FCs because the organisms self-regulate and regenerate the enzymes.

When converting biomass into fuel, different processes and microorganisms may be called for, depending on the biomass source and the processing goal [9]. For example, methanogenic species are capable of digesting a wide range of substrates but have a slow metabolism and are not robust to shock [1]. In contrast, ethanol-producing yeast (especially *Saccharomyces cerevisiae*) are hardy and capable of rapid fermentation but are limited in the substrates that can be digested [2,10]. Because of this rapid metabolism, yeast have been extensively used to produce ethanol from crops such as corn and sugar cane, but there is also considerable interest in developing alternative feedstocks [4,11]. On the processing side, a wide range of standard separation techniques (e.g., distillation, sorbents, pervaporation) have been used to concentrate the fuels and remove unwanted byproducts [2,12,13]. There has also been considerable interest in using fuel cells (FCs) with biofuels, especially ethanol [14] due to the ability of FCs to run on dilute biofuel streams, and depending on the source, with minimal parasitic power losses due to purification [15,16]. FCs also have much higher electrical power densities than MFCs (typically 3 orders of magnitude), which greatly reduces the required size for a given fuel stream.

Microbial consortia have many advantages over pure cultures and, indeed, natural consortia already find widespread use in the treatment of waste water [1,9]. In addition to these natural consortia, there is considerable interest in engineering consortia to overcome the disadvantages associated with single organism cultures. In particular, yeast (especially *S. cerevisiae*) is an attractive target for developing engineered consortia. It can rapidly ferment many simple sugars. It produces high concentrations of ethanol. It is hardy, tolerating a wide range of growth conditions. Yeast has also been intensively studied, and is reasonably well understood. Thus, there is considerable interest in pairing yeast with other organisms that can break down complex substrates into sugars that the yeast can ferment. This is especially true for cellulosic materials, where the extensive and energy-intensive preprocessing required has promoted interest in using cellulose digesting molds [11,17] or in expressing mold enzymes in yeast [18]. While preprocessing starches is easier than cellulose, it is still energy intensive and there are potential benefits to digesting it using consortia [4,10]. An ancient example of using consortia to digest starches is found in sake brewing, where the mold *Aspergillus oryzae* is used to digest rice into substrates that the yeast *S. cerevisiae* can ferment to ethanol [19]. *A. oryzae* has also been used in the fermentation of many other substrates, such as soy beans [20] and sweet potatoes [21], and has long been of interest as a source of secretory enzymes [22,23,24]. Consortia of yeast and *A. oryzae* have already been highly adapted to fermenting food into a useful fuel and therefore this consortium has naturally been considered for optimization for fuel production [4,21].

Here we demonstrate that consortia of *A. oryzae* and *S. cerevisiae* can be used to digest food waste stimulants to produce ethanol and that this fermentation can be linked with a direct alcohol FC to produce electricity from ethanol being generated from an ongoing fermentation. Aspects of preparing an *A. oryzae* and *S. cerevisiae* consortium, such as initial conditions and choice of substrates, are examined to optimize the rate of substrate digestion and the production of ethanol. Using a modified bio-hybrid FC design, ethanol is extracted continuously from the fermentation while it is being produced to allow a greater extent of substrate digestion than is possible in a batch configuration. The ethanol is then used by the FC to produce electricity at power densities typical for these direct alcohol FCs. By using this bio-hybrid FC design with consortia of *A. oryzae* and *S. cerevisiae*, electricity can be produced directly from food waste simulants without any pre- or post-processing.

## 2. Experimental Section

### 2.1. Materials

All water used in these experiments was deionized with a reverse osmosis membrane and passed through a Barstead Nanopure water polisher. Other chemicals were at least research grade and used as received from either Sigma-Aldrich or Fisher. The complex carbohydrate food simulants were rice and crackers. (These were Uncle Ben’s Converted Original Enriched Parboiled Long Grain Rice and Manischewitz Thin Unsalted Matzos. Presumably any salt-free, fat-free, preservative-free brands would give similar results.) A sterile-filtered solution of 0.5% yeast extract and 0.5% bacterial peptone, which we refer to here as 0.5% YP, provided a source of amino acids. Solution percents are weight per total volume for solids dissolved in liquids, and volume per total volume for mixtures of liquids.

### 2.2. Organisms and Culture Conditions

*A. oryzae* (RIB40) was obtained from the American Type Culture Collection (ATCC) and allowed to grow for a month on 6 cm diameter plates of 312 Czapek’s Agar. (See Supplemental Materials for the composition used.) A mixture of *A. oryzae* conidiophores (spores) and vegetative cells was obtained by flooding one plate with 25 mL of 0.5% YP and gently rubbing with the bulb end of a sterile plastic eye dropper, then vortexing the resulting mixture. This mixture was used to inoculate cultures with *A. oryzae* at a ratio of approximately 50 μL of inoculum per 1 mL of culture.

Dried *S. cerevisiae* (VL3) pellets were obtained from Laffort (Bordeaux, France) and stored at approximately 4 °C. 100 mg of these pellets were vortexed into 1 mL of 0.5% YP. This mixture was used to inoculate cultures with *S. cerevisiae* at a ratio of approximately 5 μL of inoculum per 1 mL of culture.

The cultures with soluble carbohydrate media (*i.e.*, 5% or 10% maltodextrin) were prepared by mixing appropriate amounts of sterile-filtered 15% maltodextrin, 5% YP, and water prior to inoculation. Typical culture volumes were 10 mL. In order to mimic the microaerobic conditions expected to be present with the solid substrates, these cultures were not shaken. Also, after the yeast was introduced, the culture chamber was purged daily with nitrogen after taking 50 μL liquid samples from each culture for Fourier-transform infrared spectroscopy (FTIR) analysis. Analysis of all the samples generally took about an hour, during which time the *A. oryzae* in the liquid cultures had ready access to oxygen. It continued to grow noticeably, though not abundantly, near the top of the liquid samples for at least 7 days (note that Day 0 designates the start. Day *N* is 24×*N* h later).

The cultures with solid components (e.g., crackers or rice) were prepared by loosely packing the solid substrate into a vial and then pouring liquid so that the solid substrate was mostly but not completely submerged. This incomplete covering of the solid substrate is often used to promote *A. oryzae* growth [25]. (When the solid substrates were fully submerged under 5 cm of liquid, in a preliminary experiment, *A. oryzae* growth was observed only in the liquid phase and no digestion of the solid substrate was detected by FTIR measurements.) Including the solid substrate, the typical culture volumes were 50 mL. Prior to inoculation, the rice was hydrated in hot (70 °C) water for about 40 min, then cooled to room temperature. Despite this pre-hydrating, the rice steadily absorbed water and swelled for the first 4 days, so that it remained above the liquid even as the *A. oryzae* consumed it. As with the liquid cultures, 50 μL samples of liquid were periodically taken aseptically from the “solid” cultures for FTIR analysis. No nitrogen purging was deemed necessary to maintain an anaerobic environment for the *S. cerevisiae* to ferment in, as the vials were 10 cm deep and blocked with the solid substrate. Also, by Day 4 the *A. oryzae* had covered the entire top in abundant growth.

### 2.3. Fuel Cell Measurements

The direct alcohol FCs used in this study (and in [15]) were obtained from fuelcellstore.com (SKU 1071041, H-Tec Ind., GmbH, single plate methanol/air PEMFC, 2.68 cm^2^ active area, internal impedance ≈ 10 Ω). The FCs were cleaned by soaking in 5% H_2_SO_4_ before use and after each run. The normal fuel volume that can be placed in contact with the FC’s anode is approximately 2 mL but one of the FCs was modified to have a 12 mL fuel chamber for use with an ongoing fermentation.

Amperometric (current *vs.* time) measurements of FC performance were taken with an eight channel VMP3 potentiostat obtained from Biologic (Claix, France). The potentiostat was set to hold the FCs at a constant potential of 200 mV, to match previous power optimizations [15]. The FC cathode was used as both a pseudo-reference and as the counter electrode.

### 2.4. Bio-Hybrid Fuel Cell Setup

The setup of the bio-hybrid FC was a variant of what has been previously reported in the literature [16,26]. Specifically, a Dow Filmtec SW30HR RO membrane (Sterlitech Corporation: Kent, WA, USA) was fixed between two chambers (glass half-U tubes) with a fermentation in one chamber and a 5% glucose draw solution in the other. The area of the RO membrane was approximately 8 cm^2^ and the liquid volumes were approximately 10 mL. In the case of the rice fermentation, there was also about 10 g of rice solids on the fermentation side. The draw solution was transferred periodically to a FC and the draw chamber then refilled with fresh draw solution. This setup avoids any back-diffusion of acetic acid from the FC into the fermentation. Supplemental Figure S1 shows a schematic of the entire setup.

### 2.5. Fourier Transform Infrared Spectroscopy (FTIR)

IR spectra were obtained with an Alpha FTIR spectrometer (Bruker Optics Inc.: Billerica, MA, USA) with a diamond attenuated total reflection attachment. A water background was collected immediately prior to each measurement. Sample volume was 50 μL. Each background and measurement was an average of 48 scans. To determine the composition of mixtures, the mixture spectra were fit from reference spectra of the components, using a least-squares fitting method developed in-house [27]. The methodology was tested extensively against known mixtures and compared to HPLC, and was found to have an absolute error of 0.1% or less.

## 3. Results and Discussion

*A. oryzae*–*S. cerevisiae* consortia have been used to digest both soluble starch substrates and solid substrates [19,21]. Soluble substrates are in many ways easier to study but solid substrates are very common in food and agricultural waste and are difficult to convert into soluble substrates with pre-processing [2,11,28]. Here we examine *A. oryzae*–*S. cerevisiae* consortia growing on both a simple short-chain linear soluble starch (maltodextrin) and on more complex solid carbohydrate substrates (rice and crackers). The insights obtained from the soluble substrates are used to guide the conditions for the complex substrates. Ethanol produced from the solid substrates is used in a bio-hybrid FC to generate electricity while the fermentation continues to produce ethanol.

### 3.1. Soluble Substrate (Maltodextrin)

**Figure 1:**
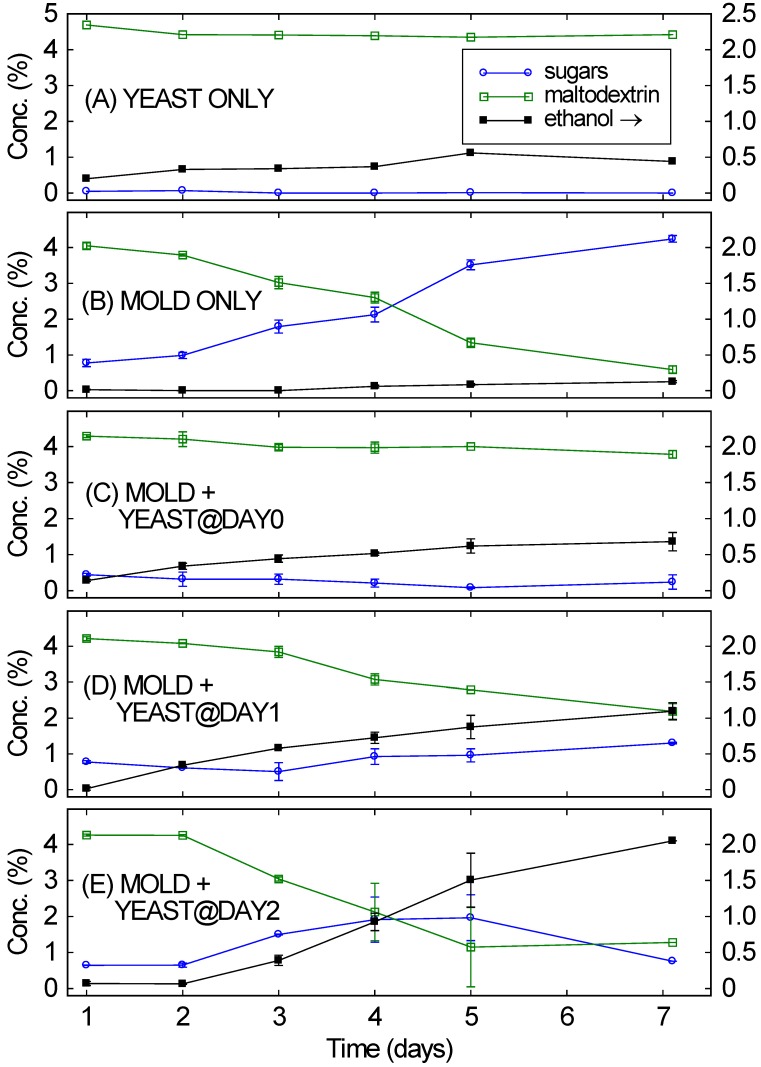
Concentrations of sugars and maltodextrin (left scale) and of ethanol (right scale) over time for 5 different consortia of the mold *A. oryzae* and the yeast *S. cerevisiae*, starting from 5% maltodextrin at Day 0. (**A**) Mold only; (**B**) Yeast only; (**C**) Mold and yeast; (**D**) Mold, with yeast added 1 day later; (**E**) Mold, with yeast added 2 days later.

Individually, *S. cerevisiae* is incapable of digesting complex substrates while *A. oryzae* does not produce ethanol. Figure 1A,B confirm that when only a single organism was added to a medium of maltodextrin, ethanol production was very limited. When only *S. cerevisiae* was present (Figure 1A), over seven days it fermented a small amount of ethanol, likely from trace amounts of simple sugars present in the maltodextrin. But its inability to break down the maltodextrin greatly limited the amount of the ethanol that was produced after seven days (0.4%). The right-hand axis’s scale is for ethanol.

When *A. oryzae* was grown by itself on the same medium (Figure 1B), the maltodextrin was nearly completely broken down into simple sugars after seven days. The rate of maltodextrin conversion into simple sugars was initially fairly slow (0.2% per day) but increased over the length of the culture, exceeding 0.5% per day by the end of the culture. Only a fraction of the broken down maltodextrin was immediately captured by *A. oryzae*. Most was available in solution as simple sugars, almost entirely maltose and maltotriose. However, ethanol was not produced in any appreciable quantities and large amounts of simple sugars remained in solution.

In contrast, when both organisms are operating properly in a consortium, they are capable of digesting complex substrates to produce ethanol. Yet not all conditions are well suited to both organisms operating in a cooperative manner. For example, as Figure 1C–E collectively show, it is beneficial to have the *A. oryzae* well-established in a medium before introducing *S. cerevisiae*. When *A. oryzae* and *S. cerevisiae* were added at the same time (Figure 1C), *A. oryzae* grew very slowly and only a small amount of the 5% maltodextrin was digested, leaving 3.8% maltodextrin after a week. No simple sugars were detected in the fermentation, likely indicating that *S. cerevisiae* converted the simple sugars to ethanol as fast as they were produced by *A. oryzae.* The fast-metabolizing yeast inhibited the mold, which reduced secretion of the mold’s enzymes, which reduced maltodextrin breakdown, which in turn inhibited the yeast. The final ethanol concentration was only 0.7%; only a little maltodextrin was digested. Similar results (not shown) were obtained if *S. cerevisiae* was added before *A. oryzae.*

Delaying the yeast gave very different results. If the *S. cerevisiae* was added one or two days after the *A. oryzae*, the *A. oryzae* had a chance to get established and to start digesting the maltodextrin before the *S. cerevisiae* began fermenting the simple sugars into ethanol. For *S. cerevisiae* added one day after *A. oryzae* (Figure 1D), the rate of maltodextrin digestion increased gradually over the fermentation, reaching slightly more than 0.3% per day by the end of the fermentation. This resulted in a decrease from 4% to 2% maltodextrin over the course of the fermentation. *S. cerevisiae* turned the simple sugars being produced by *A. oryzae* into ethanol, resulting in 1% ethanol after seven days. 

When the delay before adding *S. cerevisiae* was increased to 2 days, *A. oryzae* was even more effective in breaking down the maltodextrin, with the rate of maltodextrin breakdown exceeding 0.5% per day by the end of the fermentation. See Figure 1E. This value is similar to the rate observed without any *S. cerevisiae* present (Figure 1B), indicating that *S. cerevisiae* is not intrinsically inhibitory to *A. oryzae*. As with the other consortia, *S. cerevisiae* fermented the simple sugars into ethanol, but, since more sugars were being produced by *A. oryzae*, higher ethanol concentrations were reached (>2% by day 7). This “delayed consortium” approach has been long used in sake making [19]. Besides ensuring that *S. cerevisiae* has ample simple sugars to ferment, the delay also enables *A. oryzae* to be well-established before being stressed by the ethanol from *S. cerevisiae*. 

### 3.2. Solid Substrates (Crackers and Rice)

**Figure 2:**
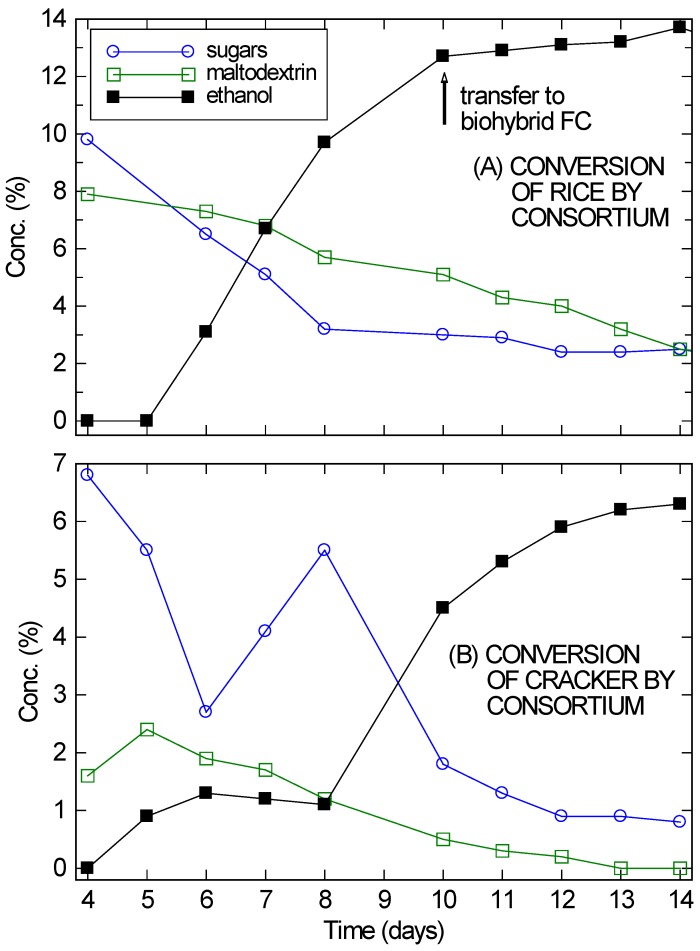
Conversion of rice (**A**) and cracker (**B**) by *A. oryzae*–*S. saccharomyces* consortium, showing the sugar, maltodextrin, and ethanol concentrations in solution, starting from Day 4 when the yeast was added. The absolute error in the concentration measurements was ±0.1%, roughly the size of the markers.

While *A. oryzae–S. cerevisiae* consortia are well suited to break down soluble starches, many substrates of interest (e.g., agricultural or food waste) are present as solids [5,11]. The hyphae produced by *A. oryzae* are particularly well suited for digesting solid substrates [25]. Two common components of food waste that are indigestible by *S. cerevisiae* were selected: crackers made with only wheat and water (here, matzo), and plain uncooked rice. When these substrates were added to 0.5% YP and inoculated with *S. cerevisiae* only, no ethanol was produced, even after several weeks, as expected (not shown). In contrast, when *A. oryzae* was added first, after four days significant amounts of simple sugars and soluble starches (fitted as maltodextrin) were observed in solution, as shown in Figure 2. On Day 4, *S. cerevisiae* was added to the culture. Four days of delay were allowed instead of the two days used with the maltodextrin to ensure that *A. oryzae* was well-established in the partially submerged solids. 

On Day 5 or 6 ethanol began to appear in significant quantities and the simple sugars and soluble starches began to drop. Over the next several days, the ethanol concentration continued to rise and the carbohydrate content in solution continued to drop. However, at the very least, enzymes from the *A. oryzae* remained active in solution and continued to digest the substrates, since the amount of ethanol being produced was much larger than the drop in sugar concentration. By Day 14, the ethanol concentration in the cracker fermentation (Figure 2B) was plateauing, even though 6.3% ethanol is not inhibitory for the VL3 strain of *S. cerevisiae* and there was still 1% unfermented sugars present in solution. For the rice culture (Figure 2A), ethanol had already reached inhibitory concentrations by Day 10.

Despite its problems, the cracker culture reached twice the ethanol concentrations needed for a direct ethanol FC. It was therefore incorporated with a modified bio-hybrid FC design [16,26] to establish that these consortium-based fermentations are compatible with use in a bio-hybrid FC. In bio-hybrid FCs, a reverse osmosis (RO) membrane allows ethanol to diffuse across, while preventing the passage of other components (e.g., peptones) that may poison the FC. For the cracker culture, the setup was allowed to fully equilibrate across the RO membrane over three days before the FC was run. Since the fermentation had mostly stopped, the ethanol concentration on the fermentation side of the RO membrane dropped from approximately 6% to 3.3%. Similarly, the ethanol concentration on the draw side rose from zero to 3.3%. The extra 0.6% ethanol was presumably from further fermentation.

**Figure 3:**
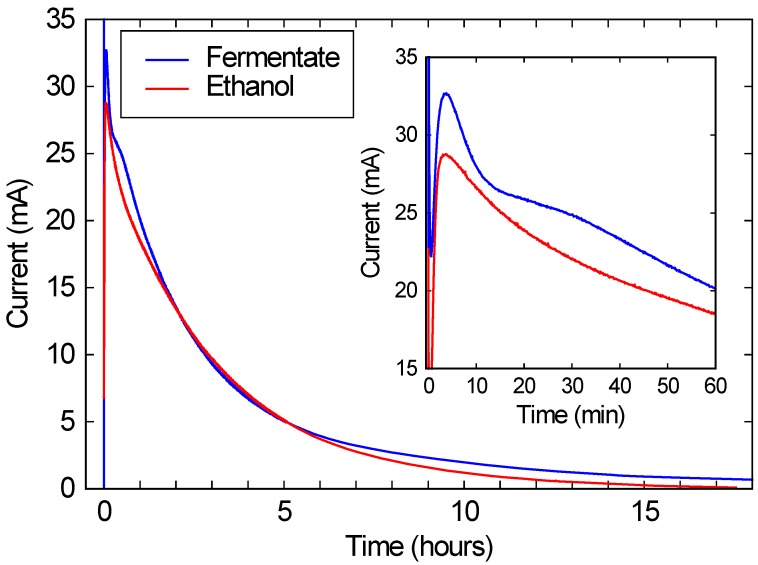
The blue line shows the current produced by a bio-hybrid fuel cell (FC), comprising a direct alcohol FC running on the draw solution of a fermentate from a consortium of *A. oryzae* and *S. cerevisiae*. The red line shows the current from the same direct alcohol FC, running on a pure ethanol/water solution with the same initial ethanol concentration (3.3%) as the draw solution. The inset expands the first hour of the run.

As shown in Figure 3, when this solution was run in a commercial FC, a peak current of over 10 mA/cm^2^ is observed, corresponding to over 2 mW/cm^2^ of power generation. (Recall that the FC area was 2.68 cm^2^, and the voltage was kept at 200 mV.) This current and power density is comparable to the typical performance for these FCs, and is roughly 3 orders of magnitude higher than power densities typically obtained with microbial fuel cells [7,15]. While the current drops substantially over a 24 h run, this drop is due only to the ethanol being consumed. As the red line in Figure 3 shows, if the same FC is run with a similar concentration of ethanol in DI water, the performance is nearly identical. These results suggest that bio-hybrid FCs can be run on these consortium-based fermentations with current and power densities comparable to FCs running on ethanol water mixtures.

**Figure 4:**
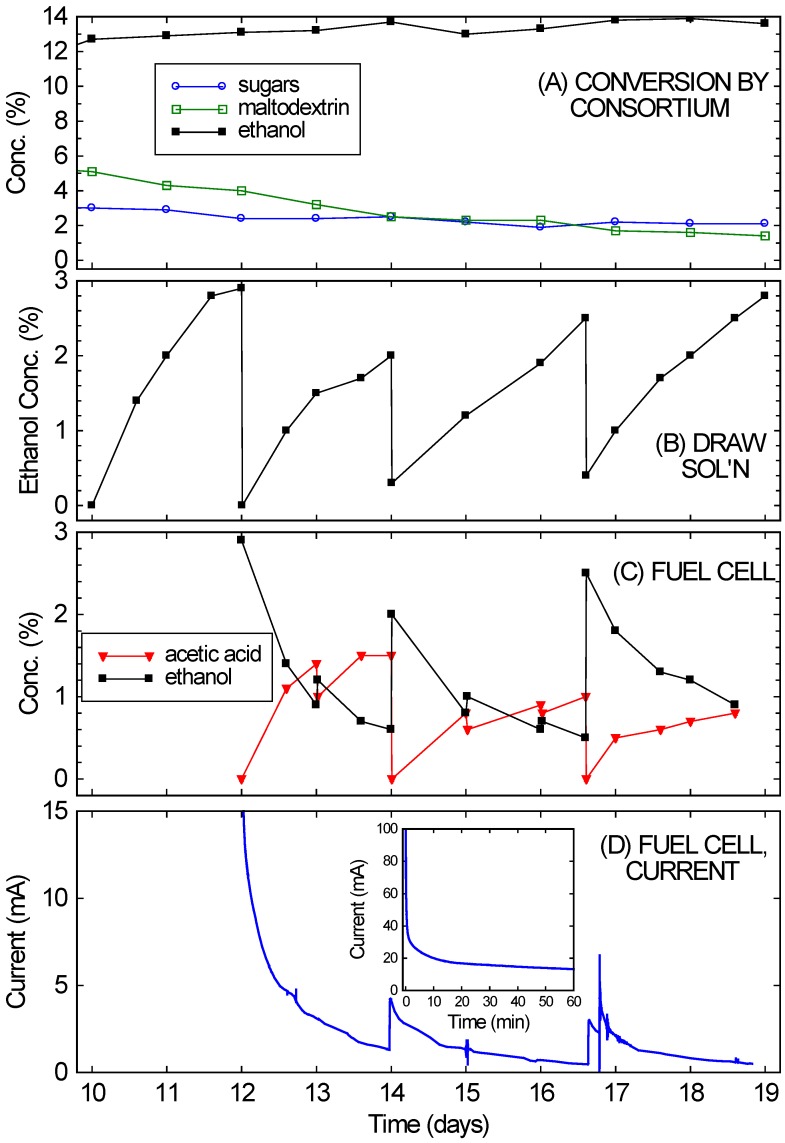
(**A**) Continued conversion of rice in the fermentation chamber of the bio-hybrid fuel cell (FC) by the *A. oryzae*–*S. cerevisiae* consortium, showing the concentrations of sugars, maltodextrin, and ethanol; (**B**) Ethanol levels in the draw solution of the bio-hybrid FC. Major transfers occurred on Days 12, 14, and 16.6; (**C**) Ethanol and acetic acid levels in the anode compartment of the FC. Minor transfers occurred on Days 13, 15, and 16; (**D**) Current generated in FC. The inset expands the first hour of the run.

Figure 4 shows that the bio-hybrid FC runs even better with an ongoing consortium-based fermentation instead of one that is dying down (as happened with the crackers). The bio-hybrid FC provides a means to draw off and use the ethanol from the consortium so it doesn’t reach concentrations that stop the yeast from fermenting. The rice culture (refer back to Figure 2A) was used for this, since it had reached a higher concentration of ethanol (13%), which was still increasing even at 10 days, but was approaching the VL3 strain’s limit of about 15%. Also, plenty of rice remained. Starting on Day 10, nearly all of the remaining liquid (10 ± 0.3 mL) and much of the remaining solids (10 ± 0.5 g) from the consortium-based fermentation of rice were transferred into the diffusion setup. This diffused across the RO membrane into a 5% glucose draw solution, while continuing to ferment. After 2 days (on Day 12), the ethanol in the draw solution had reached concentrations optimal for FC operation (2%–4%). (See Figure 4B.) The ethanol-rich draw solution was transferred into the FC, and the draw chamber was refilled with fresh draw solution. This modification of the setup in [16] prevented acetic acid produced by the FC from slowly diffusing back into the fermentation chamber and eventually killing the yeast.

The results of running the FC with the ongoing consortium-based fermentation are also shown in Figure 4. In Figure 4D, the FC currents observed over 1 week of operation (Days 12–19 of the culture) are shown (again, for voltage fixed at 200 mV). Figure 4C shows the ethanol and acetic acid concentrations in the FC anode chamber, starting with the initial fill-up with draw solution on Day 12. Over the first few days the currents were in the range expected^15^ for the ethanol concentrations. The current drop simply mirrored the ethanol drop (from consumption, evaporation, cross-over, *etc.*). At a minimum, the FC chamber was topped off daily with liquid from the draw solution. These “topping-off” transfers occurred on Days 13, 15, and 16. Every few days (Days 14 and 16.6), the FC solution was completely replaced with draw solution. By then the draw solution (Figure 4B) had reached 2%–4% ethanol, while the FC solution (Figure 4C) had low ethanol concentrations. 

Immediate current increases were observed when draw solution was added to the FC, but the currents obtained never fully returned to the initial currents. The currents observed on Day 16 were especially low compared to the expected current from the ethanol concentration. This drop in current likely arose from small amounts of sulfur-containing peptones slowly leaking across the RO membrane (from the fermentation side to the draw side) and poisoning the FC’s catalyst. Since this occurs gradually, it is feasible to alternate operation of the FC with cleaning. On Day 16.6, 5% sulfuric acid was first used to clean the FC, to try to restore its performance before refilling with draw solution. Note that this was a flush and rinse, not the standard overnight soak, so the break in current data is not noticeable on the time scale shown. After this quick cleaning, the currents returned to the values observed after the refilling with draw solution on Day 14. Alternating between two FCs, with one running and one being cleaned, would probably improve long-term performance substantially. Despite this drop in performance, the fuel cell still managed to achieve an average power density of 0.2 mW/cm^2^ over one week of operation, approximately 10% of the peak power density of these fuel cells. This power density also remains much higher than what could achieved than what could be achieved with a microbial fuel cell.

While the FC was running and ethanol diffusing across the RO membrane, the rice culture continued to produce ethanol, and in large amounts (equivalent to an additional 10% ethanol). As shown in Figure 4A, the ethanol concentration in the culture actually rose slightly (gaining another 1%) over the week it was in contact with the draw solution. The draw volume was similar to the rice culture volume, and drew off 9% ethanol. Therefore, if no fresh ethanol were being fermented in the culture, the ethanol concentration would have necessarily dropped significantly (as was observed with the dying cracker culture). Further evidence for the continued activity of the *S. cerevisiae* is the gradual drop in the sugar concentration over time. The viability of *A. oryzae* is unknown from our data. The high ethanol concentrations could have been inhibiting its growth. It is clear, however, that at least the secreted *A. oryzae* enzymes retained considerable activity. The maltodextrin-like component of the carbohydrate peaks decreased over time, which would not have occurred if only the yeast had been active. Also, the 4% combined drop in maltodextrin and sugar is not enough to explain the production of 10% ethanol. It would only account for 2% ethanol—unless it were being continually produced from the rice. The presence of these enzymes prolonged the lifetime of the fermentation, but for indefinite operation it would be desirable to maintain the viability of the *A. oryzae,* either by running the fermentation at lower concentrations or by taking steps to reduce the exposure of *A. oryzae* to the fermentation solution. Even without these precautions, the consortium-based rice fermentation can be operated for several weeks if coupled to a bio-hybrid FC to selectively remove ethanol. This allows more complete digestion of the substrate (rice in the case) and more ethanol production than would be possible in a simple batch configuration while simultaneously generating electricity at power densities approaching those obtained operating on pure ethanol water mixtures.

## 4. Conclusions

In this paper we have examined the interactions between *A. oryzae* and *S. cerevisiae* when working as a consortium and how this consortium can be used to digest food waste stimulants. If properly established in the growth medium, *A. oryzae* can break down a wide range of substrates to produce simple sugars that the yeast can convert into ethanol. We have demonstrated that bio-hybrid FCs can be used to extract this ethanol with a reverse osmosis membrane and then produce electricity. The bio-hybrid FC was operated continuously for 1 week of operation and produced power densities typical for direct alcohol FCs, although performance was declining by the end of the week. Furthermore, by operating the FC with an ongoing fermentation, it is possible to convert more of the substrate into ethanol than would be possible in an uncoupled fermentation. Overall, these results provide insights into operating fermentations with consortia of microorganisms and demonstrate how coupling biological systems with power production can not only generate electricity but also enhance microbial metabolism.

## Supplementary Materials

Supplementary materials can be found at: www.mdpi.com/2076-2607/4/1/10/s1, Figure S1: Overall Schematic of Modified Bio-Hybrid Fuel Cell Setup, showing the components and the processing steps to go from complex carbohydrates to electrical power. The components and the processing steps are described in the text. The components (blue and black) operated entirely passively. The transfers (indicated by red lines and arrows) were done manually, for experimental simplicity.
