# Supplementary material for: Aspergillus oryzae–Saccharomyces cerevisiae Consortium Allows Bio-Hybrid Fuel Cell to Run on Complex Carbohydrates
Source: Microorganisms. 2016 Feb 4;4(1):10. doi: 10.3390/microorganisms4010010 (PMC5029515; doi:10.3390/microorganisms4010010)
Supplement: Supplementary file 1 [file microorganisms-04-00010-s001.docx]

**Supplementary Materials: *Aspergillus
oryzae*–*Saccharomyces cerevisiae* Consortium
Allows Bio-Hybrid Fuel Cell to Run on
Complex Carbohydrates**

Justin P. Jahnke, Thomas Hoyt, Hannah M. LeFors, James J. Sumner and David M. Mackie

**Preparation Instructions for 312 Czapek’s Agar**

1. Mixture A:

3 g NaNO_3_

1 g K_2_HPO_4_

0.5 g MgSO_4_·7H_2_O

0.5 g KCl

0.01 g FeSO_4_·7H_2_O

15 g agar

900 mL DI water

1. Autoclave Mixture A at 121 °C. Let cool to about 50 °C.
2. Mixture B:

30 g sucrose

100 mL DI water

1. Sterile-filter Mixture B.
2. Combine Mixture A with Mixture B.
3. Pour into plates, slants, *etc*. while still warm. It will set over several hours.


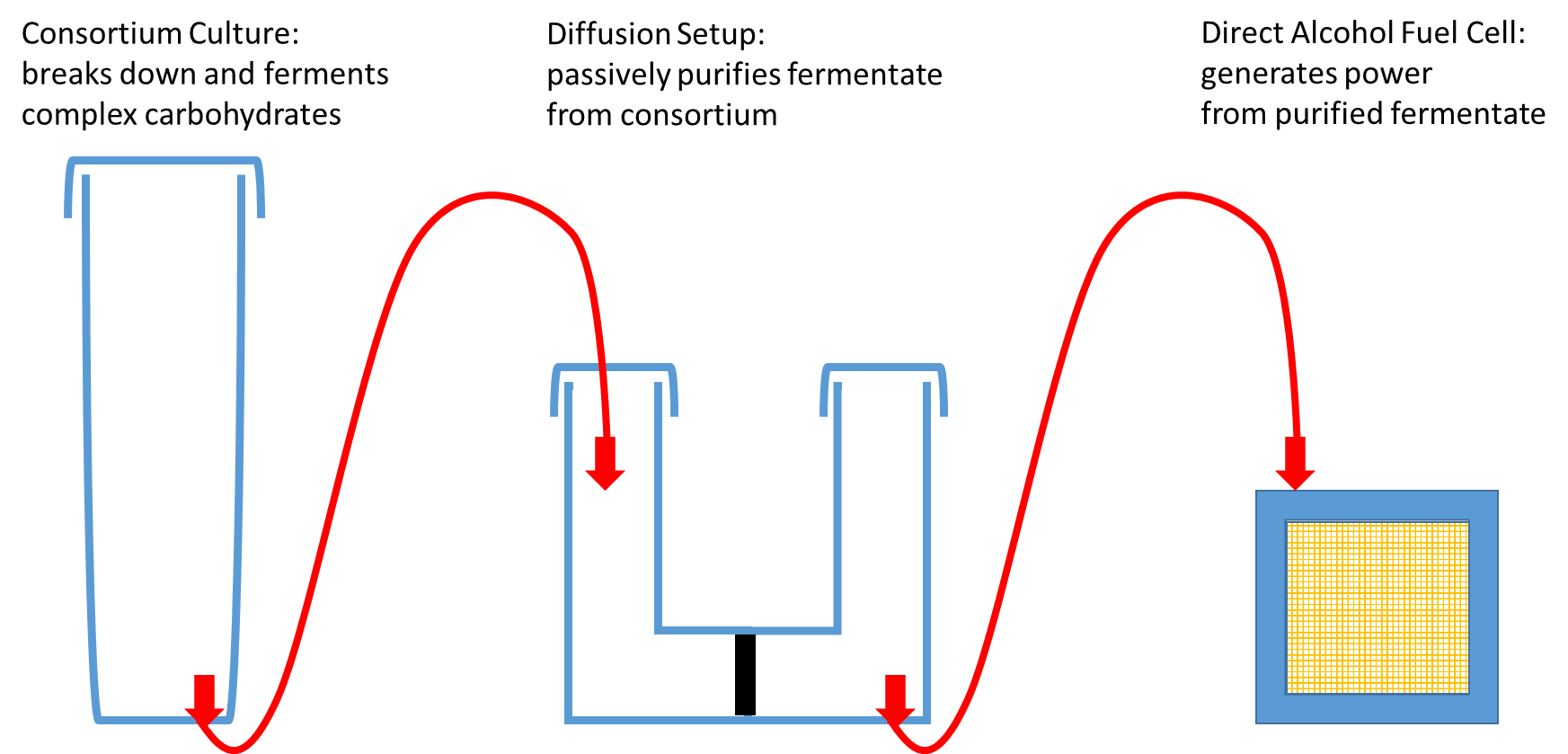


**Figure S1.** Overall Schematic of Modified Bio-Hybrid Fuel Cell Setup, showing the components
and the processing steps to go from complex carbohydrates to electrical power. The components
and the processing steps are described in the text. The components (blue and black) operated
entirely passively. The transfers (indicated by red lines and arrows) were done manually,
for experimental simplicity.

© 2016 by the authors; licensee MDPI, Basel, Switzerland. This article is an open access article distributed under the terms and conditions of the Creative Commons by Attribution (CC-BY) license (http://creativecommons.org/licenses/by/4.0/).
